# Supplementary material for: Clinicopathological and prognostic significance of caveolin-1 and ATG4C expression in the epithelial ovarian cancer
Source: PLoS One. 2020 May 13;15(5):e0232235. doi: 10.1371/journal.pone.0232235 (PMC7219755; doi:10.1371/journal.pone.0232235)
Supplement: S1 Table — (DOCX) [file pone.0232235.s001.docx]

**Table S1. Clinicopathologic features of 95 epithelial ovarian cancer patients with high or low expression of CAV1 and ATG4C in cancer cells.**

| Parameters | N | High expression of CAV1 and ATG4C | | |  | Low expression of CAV1 and ATG4C | | |
| --- | --- | --- | --- | --- | --- | --- | --- | --- |
|  |  | Yes (%) | No (%) | *P* value |  | Yes (%) | No (%) | *P* value |
| Age |  |  |  | 0.635 |  |  |  | <0.999 |
| ﹤60 years | 77 | 38(40.0) | 39(41.1) |  |  | 15(15.8) | 62(65.3) |  |
| ≧60 years | 18 | 10(10.5) | 8(8.4) |  |  | 4(4.2) | 14(14.7) |  |
| Histologic subtype |  |  |  | 0.433 |  |  |  | 0.079 |
| Serous | 47 | 26(27.4) | 21(22.1) |  |  | 5(7.4) | 42(42.1) |  |
| Clear cell | 1 | 1(1.1) | 0(0.0) |  |  | 0(0.0) | 1(1.1) |  |
| Mucinous | 17 | 9(9.5) | 8(8.4) |  |  | 6(6.3) | 11(11.5) |  |
| Endometrioid | 30 | 12(12.6) | 18(18.9) |  |  | 8(15.8) | 22(15.8) |  |
| Histologic grade |  |  |  | 0.982 |  |  |  | 0.474 |
| I | 21 | 11(11.6) | 10(10.5) |  |  | 6(6.3) | 15(15.8) |  |
| II | 28 | 14(14.7) | 14(14.7) |  |  | 6(6.3) | 22(23.2) |  |
| III | 46 | 23(24.2) | 23(24.2) |  |  | 7(7.4) | 39(41.1) |  |
| Tumor size (T) |  |  |  | 0.656 |  |  |  | 0.945 |
| T1 | 47 | 23(24.2) | 24(25.3) |  |  | 10(10.5) | 37(38.9) |  |
| T2 | 24 | 14(14.7) | 10(10.5) |  |  | 4(4.2) | 20(21.1) |  |
| T3 | 24 | 11(11.6) | 13(13.7) |  |  | 5(5.3) | 19(20.0) |  |
| Lymph node metastasis (N) | |  |  | 0.677 |  |  |  | 0.596 |
| N0 | 89 | 44(46.3) | 45(47.4) |  |  | 17(17.9) | 72(75.8) |  |
| N1 | 6 | 4(4.2) | 2(2.1) |  |  | 2(2.1) | 4(4.2) |  |
| Distant metastasis (M) | |  |  | 0.426 |  |  |  | <0.999 |
| M0 | 74 | 39(41.1) | 35(36.8) |  |  | 15(15.8) | 59(62.1) |  |
| M1 | 21 | 9(9.5) | 12(12.6) |  |  | 4(4.2) | 17(17.9) |  |
| FIGO stage |  |  |  | 0.656 |  |  |  | 0.945 |
| I | 47 | 23(24.2) | 24(25.3) |  |  | 10(10.5) | 37(38.9) |  |
| II | 24 | 14(14.7) | 10(10.5) |  |  | 4(4.2) | 20(21.1) |  |
| III/IV | 24 | 11(11.6) | 13(13.7) |  |  | 5(5.3) | 19(20.0) |  |

Data were expressed as count and percentage for categorical variables and analyzed by Chi-square test, Continuity correction, or Fisher's exact test.
