# Supplementary material for: Clinicopathological and prognostic significance of caveolin-1 and ATG4C expression in the epithelial ovarian cancer
Source: PLoS One. 2020 May 13;15(5):e0232235. doi: 10.1371/journal.pone.0232235 (PMC7219755; doi:10.1371/journal.pone.0232235)
Supplement: S2 Table — (DOCX) [file pone.0232235.s002.docx]

Table S2. **Clinicopathologic features of 79 epithelial ovarian cancer patients with high or low expression of CAV1 and ATG4C in the stroma.**

| Parameters | N | High expression of CAV1 and ATG4C | | |  | Low expression of CAV1 and ATG4C | | |
| --- | --- | --- | --- | --- | --- | --- | --- | --- |
|  |  | Yes (%) | No (%) | *P* value |  | Yes (%) | No (%) | *P* value |
| Age |  |  |  | >0.999 |  |  |  | 0.462 |
| ﹤60 years | 64 | 15(19.0) | 49(62.0) |  |  | 28(35.4) | 36(45.6) |  |
| ≧60 years | 15 | 4(5.1) | 11(13.9) |  |  | 5(6.3) | 10(12.7) |  |
| Histologic subtype |  |  |  | **0.001*** |  |  |  | 0.079 |
| Serous | 42 | 15(19.0) | 27(34.2) |  |  | 15(19.0) | 27(34.2) |  |
| Mucinous | 14 | 4(5.1) | 10(12.7) |  |  | 4(5.1) | 10(12.7) |  |
| Endometrioid | 23 | 0(0.0) | 23(29.1) |  |  | 14(17.7) | 9(11.4) |  |
| Histologic grade |  |  |  | 0.586 |  |  |  | 0.153 |
| I | 18 | 5(7.1) | 13(18.6) |  |  | 5(6.3) | 13(16.5) |  |
| II | 23 | 8(11.4) | 15(21.4) |  |  | 8(10.1) | 15(19.0) |  |
| III | 38 | 6(8.6) | 23(32.9) |  |  | 20(25.3) | 18(22.8) |  |
| Tumor size (T) |  |  |  | 0.750 |  |  |  | 0.981 |
| T1 | 38 | 8(10.1) | 30(30.8) |  |  | 16(20.3) | 22(27.8) |  |
| T2 | 21 | 5(6.3) | 16(20.3) |  |  | 9(11.4) | 12(15.2) |  |
| T3 | 20 | 6(7.6) | 14(17.7) |  |  | 8(10.1) | 12(15.2) |  |
| Lymph node metastasis (N) | |  |  | >0.999 |  |  |  | 0.644 |
| N0 | 74 | 18(22.8) | 56(70.9) |  |  | 30(30.8) | 44(55.7) |  |
| N1 | 5 | 1(1.3) | 4(5.1) |  |  | 3(3.8) | 2(2.5) |  |
| Distant metastasis (M) | |  |  | 0.915 |  |  |  | 0.778 |
| M0 | 61 | 14(17.7) | 47(59.5) |  |  | 26(32.9) | 35(44.3) |  |
| M1 | 18 | 5(6.3) | 13(16.5) |  |  | 7(8.9) | 11(13.9) |  |
| FIGO stage |  |  |  | 0.750 |  |  |  | 0.981 |
| I | 38 | 8(10.1) | 30(30.8) |  |  | 16(20.3) | 22(27.8) |  |
| II | 21 | 5(6.3) | 16(20.3) |  |  | 9(11.4) | 12(15.2) |  |
| III/IV | 20 | 6(7.6) | 14(17.7) |  |  | 8(10.1) | 12(15.2) |  |

Data were expressed as count and percentage for categorical variables and analyzed by Chi-square test, Continuity correction, or Fisher's exact test. ^*^indicates that the difference was statistically significant.
