# Supplementary material for: Clinicopathological and prognostic significance of caveolin-1 and ATG4C expression in the epithelial ovarian cancer
Source: PLoS One. 2020 May 13;15(5):e0232235. doi: 10.1371/journal.pone.0232235 (PMC7219755; doi:10.1371/journal.pone.0232235)
Supplement: Attachment — Submitted filename: Response to Reviewers.docx [file pone.0232235.s005.docx]

**Dear Dr.** **Ivan R. Nabi:**

Thank you very much for your letter and good advices on our manuscript entitled “Clinicopathological and prognostic significance of caveolin-1 and ATG4C expression in the epithelial ovarian cancer”, with the manuscript ID number PONE-D-20-00029. We have revised the manuscript, and will resubmit it for your consideration. We have carefully studied and addressed the comments raised by the academic editor and reviewer, and have made the corrections highlighted in red for our revised manuscript. Point by point responses to the reviewer’s comments are listed below this letter.

We hope the revised manuscript is acceptable for publication in your journal. We look forward to hearing from you regarding our submission. And we would be glad to respond to any further questions and comments that you may have.

We would like to express our sincere thanks to the reviewers for the constructive and positive comments.

**Replies to Reviewer #1:**

Specific Comments

1. The authors should expand the analysis to include additional sources of data (sequencing as well as tissue). They should also consider correlating Cav1 and ATG4C and confirming IHC findings by performing or analyzing stromal and cancer cells at a single cell resolution.

Response: On the first comment, we understand that deeper experiments including sequencing and tissue may better reveal the expression pattern of CAV1/ATG4C mRNA and protein in epithelial ovarian cancer. However, in the present study, we mainly focused on the prognostic value of CAV1/ATG4C, and we think that the bioinformatics analysis of genes and immunohistochemistry analysis of proteins to detect the CAV1/ATG4C expression levels may not be optimal, but should be sufficient to draw a conclusion that reduced CAV1 levels and increased ATG4C levels associate with a poorer prognosis. And we have added this limitation to the Discussion (page 18, paragraph 1, line 430-432) in the revised version. On the second comment, the analysis of correlation between CAV1 and ATG4C have been added in the Results (page 13, paragraph 1, line 319-327, Table 3) and Discussion (page 17, paragraph 2, line 409-411 and line 420-423) in the revised version. On the third comment, we conducted IHC analysis at tissue level and distinguished cancer cells from stromal cells based on cell morphology in the microscopic high power field in the present study. Further studies at cell level, such as single cell sequence, Western blot and polymerase chain reaction (PCR) assays will be conducted to confirm the IHC findings in the future. Right now, COVID-19 outbreak in our city, Wuhan. All the labs in our University have to be closed.

2. The stromal alterations in Cav1 should be confirmed algorithmically in large patient data sets.

Response: We accept your suggestions on the sample size of ovarian cancer stroma specimen. There are some explanations for this issue. On the one hand, since some patients were reluctant to undergo resection of ovarian cancer, we collected 95 ovarian cancer samples. 16 of these samples, which contained so few stromal compartments to evaluate the immunoreactive score, were excluded from the algorithm of the stromal alterations in CAV1. On the other hand, although the sample size was slightly smaller, the results exhibited decreased stromal CAV1 expression compared with noncancerous ovarian tissues, which is consistent with the majority of evidence (please see Ref.18, Ref.19 and Ref.38). And we have taken the limited stromal sample size as a deficiency in the Discussion (page 18, paragraph 1, line 428-429) in the revised version, will detect more samples of EOC in the future.

3. Some mechanistic insight should be provided. How are these genes altered?

Response: It’s good advice. Several sentences about mechanistic insight into genes alteration have been added in Discussion (page 15, paragraph 1, line 351-355) to address this issue. During COVID-19 time, our lab closed about two months, maybe will open in the May. We always focus on CAV1 functions in the development of cancer, and published a few papers.

Other changes:

a. The citation to ‘**Table 1**’ should have been ‘**Table 2**’ in line 161. The citation to ‘**Table 3 and Table 4**’ should have been ‘**Table 4 and Table 5**’ in line 333.
b. The duplicated text outside the methods section have been rephrased in the revised version, especially in the Introduction and Discussion section.
c. Some minor formation revision has been made in the revised version according to the PLOS ONE style templates, including the location of figures captions and tables, the figure citations, line number, etc.
d. Authors’ affiliations have been amended in the title page in the revised version.

By the way, we published one paper about Caveolin-1 in the gastric cancer in your journal 2013, it has been cited about 60 times, and gained top 10% cited in the 2017. So we believe this paper may be of particular interest to the readers of your journal.

Best wishes,

Hong-Lei Chen, MD., Ph.D.

Department of Pathology

School of Basic Medical Sciences

Wuhan University

185 Donghu Road, Wuchang District

Wuhan, Hubei 430071

Office: 86-27-67811732

Fax: 86-27-68759310

Email: hl-chen@whu.edu.cn
