# Supplementary material for: Clinicopathological and prognostic significance of caveolin-1 and ATG4C expression in the epithelial ovarian cancer
Source: PLoS One. 2020 May 13;15(5):e0232235. doi: 10.1371/journal.pone.0232235 (PMC7219755; doi:10.1371/journal.pone.0232235)
Supplement: Attachment — Submitted filename: Response to Reviewers.docx [file pone.0232235.s006.docx]

**Dear Dr.** **Ivan R. Nabi:**

Thank you very much for your letter and for the comments concerning our manuscript entitled “Clinicopathological and prognostic significance of caveolin-1 and ATG4C expression in the epithelial ovarian cancer” (The manuscript ID number: PONE-D-20-00029). Those comments are all valuable and very helpful for revising and improving our paper, as well as the important guiding significance to our researches. We have studied comments carefully and have made a necessary correction which we hope meet with approval. Revised portion is marked in red in the revised manuscript. The main corrections in the paper and the response to the reviewer’s comments are detailed below this letter.

We hope the revised version of the manuscript is acceptable for publication in your journal. We look forward to hearing from you regarding our submission. If you have any inquiries, please don’t hesitate to contact me at the address below.

Thanks again for your comments and suggestions.

With best wishes,

Yours sincerely,

Hong-Lei Chen, MD., Ph.D.

Department of Pathology

School of Basic Medical Sciences

Wuhan University

185 Donghu Road, Wuchang District

Wuhan, Hubei 430071

Office: 86-27-67811732

Fax: 86-27-68759310

Email: hl-chen@whu.edu.cn

**Replies to the Reviewer:**

Specific Comments

1. The presentation of a significant correlation between CAV1 and Atg4C in tumor and stromal cells in Table 3 is particularly interesting. It would be important therefore to present the clinicopathological features of not only high vs low CAV1 and high vs low ATG4C (Tables 4 and 5) but more particularly whether the 48 high CAV1 and high ATG4 tumors or low CAV1/low ATG4 stroma present distinctive clinicopathological features.

Response: We truly appreciate the reviewer’s suggestion, and we recognize that it would greatly improve the scientific level of our manuscript. We have additionally analyzed the statistical correlations between the high or low expression of both CAV1 and ATG4 in tumors or stroma and the clinicopathologic features, including age, histologic subtype, histologic grade, T, N, M, and FIGO stage of EOC. And we found that high expression of both CAV1 and ATG4C protein in the stroma was significantly related to the histologic subtypes of EOC patients (P=0.001). We have added these results and interpretations to the Results (page 14, paragraph 1, lines 291-295, Table 6 and Table 7) and Discussion section (page 17, paragraph 2, lines 329-331; page 20, paragraph 2, lines 397-399) in the revised version of the manuscript.

Other changes:

a. The explanation of the asterisk and the description of data processing in the footnote of Table 3, 4, and 5 were rephrased in the revised version of the manuscript.
b. Some minor corrections of spelling mistakes were included in the revised version of the manuscript; all marked in red, for example, the word “endometrioid” on page 1 line 38, “significant” on page 1 line 39, “an” on page 1 line 46, “recycling” on page 4 line 73, “further” on page 4 line 81, etc.
